# On the Weight and Specific Gravity of the Brain in Insanity

**Published:** 1854-04-01

**Authors:** David Skae

**Affiliations:** PHYSICIAN TO ROYAL EDINBURGH LUNATIC ASYLUM


### ON THE WEIGHT AND SPECIFIC GRAVITY OF THE BRAIN IN INSANITY.

BY DAVID SKAE, M.D., PHYSICIAN TO ROYAL EDINBURGH LUNATIC ASYLUM.

Dr. SKAE commenced by remarking on the obscurity in which the pathology of insanity was involved, notwithstanding the careful examinations of the brain in those affected with the disease which have been carried on for many years in our principal asylums.

Such morbid appearances a sthickening and opacity of the arachnoid, serous effusions into the subarachnoid tissue, arachnoid sac and ventricles, increased or diminished vascularity of brain and membranes, were found very frequently in persons dying of other diseases and without any mental affection; while, on the other hand, cases were not unfrequently met with of patients who had exhibited during life, for many months or even years, all the symptoms of raving madness, and in whose brains the appearances described above were altogether wanting, and nothing abdnormal could be detected .A solitary exception to this general statement had been adduced in the special form of disease called general paralysis, where a peculiar softening of the grey matter was found, not indicated by any change appreciable to the eye, but by layers of the grey matter stripping off readily with the membranes, to which it often adhered; by the readiness with which it was broken up by a stream of water; and by an increase in the size of the nucleated cells of which the grey matter is principally composed. It must be inferred, therefore, that such appearances, though frequent concomitants, did not constitute the essential pathological conditions of the disease. Indeed, the analogy of the symptoms to those produced by poisons which are known to enter the blood, the suddenness of the invasion in some cases, and the suddenness of the cure in others—even occasionally of long standing; the remarkable remissions, and the temporary restoration to perfect sanity, might well lead us to doubt whether morbid changes in the structure of the brain would ever be discovered, and to look upon insanity as a disease of the blood, or due to some peculiar orgasm of the nervous centres not to be recognised after death.

It had occurred to Dr. Skae some years ago, that it might be useful to collect the weights of the brain, cerebrum and cerebellum; to measure the depth of the grey matter; to endeavour to determine the degree of hardness or softness of the cerebral substance; to ascertain by what length of a column of water it could be broken up; and, more lately, to take the specific gravity of the grey and white substance of different parts of the brain. In this communication, however, he would confine himself to the weight and specific gravity of the brain, leaving the other items of his investigation till such time as his data accumulated, to enable him to present them in a satisfactory form.

His observations on the weights of the brain were compared, in elaborate tables, with the data collected in the Royal Infirmary of

Edinburgh, by the late Dr. John Reid and Dr. Peacock, and his experiments on the specific gravity with those published by Dr. Sankey in the *Med.-Chirurg. Rev.* for January 1853, and made in the Royal London Fever Hospital. The weights used were avoirdupois.

*Weights.*—One hundred and seventy-two cases were examined, of whom eighty-three were males and eighty-nine females. In only one instance did the encephalon exceed 60 ounces in the insane, while in several of the sane it amounted to 62 ounces and upwards; the heaviest brain in the one series being 60 oz. 8 dr., and the heaviest in the other being 62 oz. 12 dr. With two exceptions, the superiority in weight was among the brains of the sane in each of the quinquennial or decennial periods into which they were classified. Dr. S. could not venture to say whether this justified the inference that persons having large brains were less frequently the subjects of mental derangement than others, but the fact was a striking one when contrasted with the results derived from a comparison of the average weight of the entire number of cases, where it appeared that the average weight was increased in persons dying insane. The average weight in the insane, from fifteen to ninety years of age, being 50 oz. 4 dr., and in the sane 49 oz. 14 dr. The same results were derived from a comparison of the weights of the brains of females. On taking the average of all the cases, the weight of the brain in the insane was 44 oz. 7 dr., and in the sane 44 oz. 5 dr. The presumption that the absolute weight of the brain is increased in the insane was greatly strengthened by the fact, that in many cases of insanity the absolute size of the brain must be materially diminished by the large quantity of serous effusion generally met with. The cerebellum, however, appeared to be the chief agent in determining this increase in weight, for on comparing the weights of the cerebra in the two series of cases the difference was inconsiderable, and indeed, in the case of the females, in favour of the sane; while on comparing the weight of the cerebellum with the pons and medulla in the two classes, it would be found that there is almost a uniform preponderance in the weights of those of the insane. The average weight in all the cases of the insane males exceeds that of the sane by four drachms, and the females by three drachms. The same fact was very clearly brought out on comparing the ratio between the cerebellum (with the pons and medulla) and the cerebrum at the different ages distinguished in the tables.

On arranging the weights of the brain according to the form of disease under which the patients laboured, and taking the average, the diseases stood in the following series: mania, monomania, dementia, and general paralysis. The weight being greatest in mania, and least in general paralysis. On comparing the average weights of the cerebella, however, the series stands thus: general paralysis, mania, monomania, and dementia; the cases of general paralysis presenting the highest average. The increase of the relative weight of the cerebellum to the cerebrum appearing to bear a constant relation to the form of the disease, and to be greatest in the more protracted and gravest cases. In acute mania, a disease of comparatively short duration, there was the smallest amount of increase in the relative weight of the cerebellum, and in general paralysis the greatest increase took place.

*Specific Gravity.*—The elaborate tables exhibited went to show that the specific gravity in the cases of insanity was almost uniformly higher, and this applied to both the grey and white matters. As regards the white matter, the mean of all the cases was 1041·1 in the sane, and 1042·4 in the insane, showing an increase in the specific gravity in cases of insanity. These results were corroborative of those obtained by Dr. Sankey, at the London Fever Hospital, who found that in all the cases complicated with cerebral symptoms of a grave character preceding death, the specific gravity was high, the average being 1043.

Dr. Skae found that in most of those cases where the specific gravity of the grey matter was considerably below the mean, the patients had died of phthisis, and in other instances of exhaustion occurring at an advanced age. In a few exceptional cases, either the symptoms immediately preceding death were of a grave character, or the morbid appearances found in the membranes indicated chronic inflammatory action.

The number of observations with regard to the cerebellum was too few to warrant more than a presumption that the specific gravity of the cerebellum was higher than that of the cerebrum.

In the different forms of mental disease, and taking the average in all the cases of each kind, the lowest specific gravity of the grey matter occurred in cases of dementia; where, however, it was still ·003 above the average in the sane. The next highest specific gravity occurred in cases of melancholia and monomania; the next in general paralysis; the next in mania; and the highest in epilepsy. Of the white matter, the lowest average specific gravity occurred in cases of mania; the next in dementia; the next highest in general paralysis; the next in monomania; and the highest in epilepsy.

Dr. Skae apologized for the meagreness of the facts presented to the Society, but they were all which he had been able to collect in the hospital under his charge. The inferences from them appeared to him of sufficient interest to warrant the hope that more extended observations of a similar kind might lead to satisfactory and important deductions.

In answer to queries from the President and Dr. Bennett, Dr. Skae said that a common scalpel was the instrument employed to separate the white from the grey matter; and that the specific gravities were obtained according to the plan proposed by Dr. Sankey, viz., by means of saline solutions of different strengths. The precaution must be taken to remove the piece of brain as soon as possible, and not to allow it to soak in the fluid.—(*Communicated to the Medico-Chirurgical Society of Edinburgh.*)

